# Editorial: Current trends in immunotherapy: from monoclonal antibodies to CAR-T cells

**DOI:** 10.3389/fmmed.2025.1633469

**Published:** 2025-06-02

**Authors:** Kishor Pant, Mark C. Glassy

**Affiliations:** ^1^ The Hormel Institute, University of Minnesota, Austin, MN, United States; ^2^ Neuro-Oncology Laboratory, Moores Cancer Center, University of California, San Diego, San Diego, CA, United States

**Keywords:** immunotherapy, monoclonal antibodies, CAR-T cells, cancer therapy, targeted therapy

Chemotherapy, radiation therapy, and surgical intervention have been the primary modalities for cancer treatment in the past few decades (Liu et al., 2024). Although these therapies often show short-term effectiveness, they exhibit many significant drawbacks, including toxicity, a lack of specificity, and the emergence of drug resistance. The scientific community is now exploring alternative ways that could provide more specific, persistent results with reduced undesirable consequences (Anand et al., 2022).

Immunotherapy is an innovative approach that has attracted significant attention and has emerged as a potent instrument in the fight against cancer (Zhang et al., 2025). The basic principle of immunotherapy is simple but effective: using the human body immune system to identify, target, and eliminate cancer and proliferating cells. This technique has the potential for sustained disease management by utilizing the immune system’s inherent ability to differentiate between healthy and aberrant cells and to adapt its response over time (Zhang et al., 2025).

Chimeric antigen receptor (CAR)-T cell therapy has transformed targeted immunotherapy, facilitating the treatment of both haematologic and solid tumours, in addition to non-oncologic disorders (Patel et al., 2025). This innovative therapy originated from years of progressive developments in cell-based therapeutics and continues to advance to address significant challenges. The progress in immunotherapy is highlighted in this Research Topic, *Current Trends in Immunotherapy: From Monoclonal Antibodies to CAR-T Cells*. It addresses progress from innovative cellular therapies like CAR-T cells to treatments based on monoclonal antibodies. The included articles illustrate how the future of immune-based treatments is being affected by developments in genetics, bioengineering, and molecular biology (Figure 1).

**FIGURE 1:**
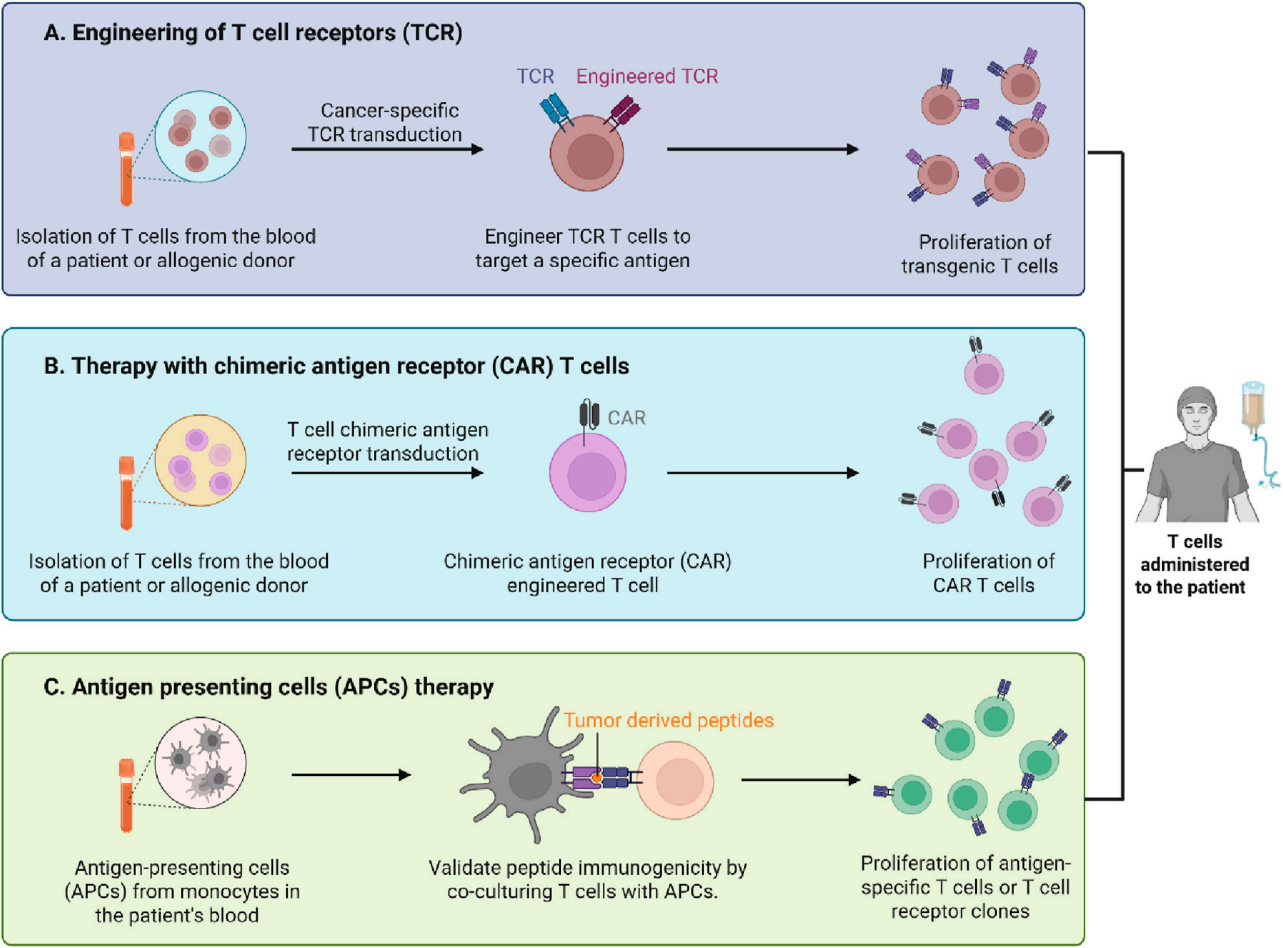
The illustration highlights 3 T cell-mediated immunotherapy approaches employed in cancer treatment. **(A)** T cell receptor (TCR) engineering involves removing T cells from a patient or donor, genetically modifying them to express cancer-specific TCRs that identify tumour antigens and subsequently proliferating and infusing them back into the patient. **(B)** Chimeric antigen receptor (CAR) T cell treatment entails the transduction of T cells with CAR constructs that facilitate antigen recognition independent of MHC presentation, resulting in the growth of CAR T cells for reintroduction. **(C)** Antigen-presenting cell (APC) treatment involves loading APCs, obtained from a patient’s monocytes, with tumor-derived peptides and co-culturing them with T cells to verify their antigen specificity, resulting in the proliferation of antigen-specific T cells or TCR clones (Prepared using BioRender https://app.biorender.com/).

A variety of investigations focus on the interactions between immune cells and cancer cells, providing new insights into how tumors evade immune identification and strategies to counteract these evasion mechanisms (Wu et al., 2024). Others concentrate on the development of immune checkpoint inhibitors, which have revolutionised the management of malignancies such as renal cell carcinoma, non-small cell lung cancer, and melanoma. This chapter also addresses the increasing prominence of personalised vaccinations, oncolytic viruses, and bispecific antibodies (Khosravi et al., 2024).

CAR-T cell therapy, wherein a patient’s T cells are genetically modified to identify and target cancer, is among the most interesting and complex subjects addressed (Kirouac et al., 2023). Initially developed for haematological malignancies, research is currently broadening its use to solid tumours, addressing challenges such as antigen heterogeneity and inhibition by the tumour microenvironment (Kirouac et al., 2023).

This Research Topic of essays emphasises the progress and promise of immunotherapy as a robust and adaptable cancer treatment approach. They highlight ongoing challenges that must be addressed to fully actualise the potential of immunotherapy, including immune-related toxicities, variability in patient responses, and the substantial expense of certain medicines.

Immunotherapy is now considered a validated treatment mode. It is now strongly recognized as the fourth pillar of cancer treatment, alongside radiation, chemotherapy, and surgery (Tagliabue et al., 2018). Immunotherapy is expected to have increasing significance in personalized and precision oncology as research advances our understanding of the immune system and its interactions with cancer (Tagliabue et al., 2018). This Research Topic serves as a resource for researchers, physicians, and students, encapsulating a specific point in the growth of the field.

## References

[B1] Anand U. Dey A. Chandel A. K. S. Sanyal R. Mishra A. Pandey D. K. (2022). Cancer chemotherapy and beyond: current status, drug candidates, associated risks and progress in targeted therapeutics. Genes Dis. 10, 1367–1401. 10.1016/j.gendis.2022.02.007 37397557 PMC10310991

[B2] Khosravi G.-R. Mostafavi S. Bastan S. Ebrahimi N. Gharibvand R. S. Eskandari N. (2024). Immunologic tumor microenvironment modulators for turning cold tumors hot. Cancer Commun. lond. Engl. 44, 521–553. 10.1002/cac2.12539 PMC11110955 38551889

[B3] Kirouac D. C. Zmurchok C. Deyati A. Sicherman J. Bond C. Zandstra P. W. (2023). Deconvolution of clinical variance in CAR-T cell pharmacology and response. Nat. Biotechnol. 41, 1606–1617. 10.1038/s41587-023-01687-x 36849828 PMC10635825

[B4] Liu B. Zhou H. Tan L. Siu K. T. H. Guan X.-Y. (2024). Exploring treatment options in cancer: tumor treatment strategies. Signal Transduct. Target. Ther. 9, 175–244. 10.1038/s41392-024-01856-7 39013849 PMC11252281

[B5] Patel K. K. Tariveranmoshabad M. Kadu S. Shobaki N. June C. (2025). From concept to cure: the evolution of CAR-T cell therapy. Mol. Ther. 33, 2123–2140. 10.1016/j.ymthe.2025.03.005 40070120 PMC12126787

[B6] Tagliabue L. Capozza A. Maioli C. Luciani A. Ierardi A. M. Carrafiello G. (2018). Immunotherapy treatment: an issue for metabolic response. Q. J. Nucl. Med. Mol. Imaging Off. Publ. Ital. Assoc. Nucl. Med. AIMN Int. Assoc. Radiopharm. IAR Sect. Soc. 62, 140–151. 10.23736/S1824-4785.17.03035-7 29166752

[B7] Wu B. Zhang B. Li B. Wu H. Jiang M. (2024). Cold and hot tumors: from molecular mechanisms to targeted therapy. Signal Transduct. Target. Ther. 9, 274. 10.1038/s41392-024-01979-x 39420203 PMC11491057

[B8] Zhang M. Liu C. Tu J. Tang M. Ashrafizadeh M. Nabavi N. (2025). Advances in cancer immunotherapy: historical perspectives, current developments, and future directions. Mol. Cancer 24, 136. 10.1186/s12943-025-02305-x 40336045 PMC12057291

